# The predicted secretome and transmembranome of the poultry red mite *Dermanyssus gallinae*

**DOI:** 10.1186/1756-3305-6-259

**Published:** 2013-09-11

**Authors:** Sabine Schicht, Weihong Qi, Lucy Poveda, Christina Strube

**Affiliations:** 1 Institute for Parasitology, University of Veterinary Medicine Hannover, Buenteweg 17, 30559 Hannover, Germany; 2 Functional Genomics Center Zurich, Winterthurerstrasse 190, 8057 Zurich, Switzerland

**Keywords:** Next generation sequencing, In silico, Excretory/secretory proteins, Secreted proteins, Transmembrane proteins, Acari, Blood digestion, Embryogenesis, Ninjurin

## Abstract

**Background:**

The worldwide distributed hematophagous poultry red mite *Dermanyssus gallinae* (De Geer, 1778) is one of the most important pests of poultry. Even though 35 acaricide compounds are available, control of *D. gallinae* remains difficult due to acaricide resistances as well as food safety regulations. The current study was carried out to identify putative excretory/secretory (pES) proteins of *D. gallinae* since these proteins play an important role in the host-parasite interaction and therefore represent potential targets for the development of novel intervention strategies. Additionally, putative transmembrane proteins (pTM) of *D. gallinae* were analyzed as representatives of this protein group also serve as promising targets for new control strategies.

**Methods:**

*D. gallinae* pES and pTM protein prediction was based on putative protein sequences of whole transcriptome data which was parsed to different bioinformatical servers (SignalP, SecretomeP, TMHMM and TargetP). Subsequently, pES and pTM protein sequences were functionally annotated by different computational tools.

**Results:**

Computational analysis of the *D. gallinae* proteins identified 3,091 pES (5.6%) and 7,361 pTM proteins (13.4%). A significant proportion of pES proteins are considered to be involved in blood feeding and digestion such as salivary proteins, proteases, lipases and carbohydrases. The cysteine proteases cathepsin D and L as well as legumain, enzymes that cleave hemoglobin during blood digestion of the near related ticks, represented 6 of the top-30 BLASTP matches of the poultry red mite’s secretome. Identified pTM proteins may be involved in many important biological processes including cell signaling, transport of membrane-impermeable molecules and cell recognition. Ninjurin-like proteins, whose functions in mites are still unknown, represent the most frequently occurring pTM.

**Conclusion:**

The current study is the first providing a mite’s secretome as well as transmembranome and provides valuable insights into *D. gallinae* pES and pTM proteins operating in different metabolic pathways. Identifying a variety of molecules putatively involved in blood feeding may significantly contribute to the development of new therapeutic targets or vaccines against this poultry pest.

## Background

The poultry red mite *Dermanyssus gallinae* (De Geer, 1778) is a worldwide distributed parasitic mite of poultry. It affects its hosts by blood feeding, causing skin irritations, weight loss, restlessness, feather pecking, and an increased incidence of cannibalism [1,2]. Furthermore, in cases with a high infestation rate it may even cause death due to anemia. As a consequence, the parasite leads to high economic losses in poultry farming with estimated annual costs of €130 million throughout the European Union alone. Therefore, the poultry red mite is the major pest for poultry farming [2,3]. The prevalence of *D. gallinae* depends on flock systems: infestation rates were 4% in cage systems but 33% in alternative systems and 67% of backyard flocks [3,4]. In different countries, *D. gallinae* prevalence rates can reach up to 80-90% as shown for the United Kingdom, The Netherlands, Italy, Serbia, Montenegro, Morocco and Japan [3]. Control of the poultry red mite is extremely difficult even though 35 effective compounds of different acaricide groups such as pyrethroids or carbamates are available [2]. However, repeated or long-term chemical control may often lead to acaricide resistance of *D. gallinae*, as shown for carbaryl, permethrin, or DTT [5-8]. Increasing resistance combined with a lack of newly discovered acaricide substance groups show the importance of new development and intervention strategies to ensure animal welfare and to reduce economic losses in poultry farming. Such strategies might be targeted drug development by identifying drug targets and substance libraries screening. Alternatively, future control strategies could rely on vaccine development, which seems a feasible way to combat this hematophagous parasite. Homologous immunization of laying hens with soluble proteins extracted from *D. gallinae* achieved 50.6% mite mortality [9]. Heterologous immunization of poultry with recombinant *Rhipicephalus microplus* (formerly *Boophilus microplus*) Bm86, a membrane-bound midgut surface protein which is used as a vaccine antigen against the mentioned cattle tick, increased *D. gallinae* mortality by 23% (not significant) compared to the control group, whereas heterologous poultry immunization with recombinant subolesin originating from the mosquito *Aedes albopictus* increased *D. gallinae* mortality by 35.1% (p = 0.009) [10]. However, to date, no vaccine candidate with appropriate potential of mite control is available.

Excretory/secretory (ES) proteins play an important role in the host-parasite interface while acting as virulence factors or immune regulators to host immune recognition. Thus, they are crucial for survival of the parasite inside and outside the host organism [11,12]. As ES proteins are supposed to be involved in causing clinical infections in the host organism, they represent a favored group of antigens for the development of new therapeutical solutions e.g. as vaccine candidates or drug targets [12-14]. The current study was conducted to identify and functionally annotate putative ES (pES) and transmembrane (pTM) proteins of *D. gallinae* by *in silico* analysis of 454 pyrosequencing generated transcriptome data, which include all developmental stages of starved as well as fed mites [15]. These first analyses of the secretome as well as transmembranome of an acarid species provide potential *D. gallinae* drug targets or vaccine candidates against this major poultry pest.

## Methods

### Identification of *D. gallinae* pES and pTM proteins

*D. gallinae* pES and pTM protein identification was based on putative protein sequences of whole transcriptome data recently made available by Schicht *et al.*[15]. Those transcriptome data were generated by two 454-pyrosequencing runs of a pooled cDNA sample of all developmental stages (from egg to the adult stage) and sex of starved as well as freshly blood fed *D. gallinae* mites. Conceptual translation of the resulting 267,464 *D. gallinae* nucleotide sequences produced 55,129 (20.6%) coding regions derived from 17,860 isotigs, 24 contigs and 37,245 singletons.

*In silico* prediction of pES and pTM protein was carried out according to the protocol of Garg and Ranganathan [12], who conducted pES protein prediction by combining the computational tools SignalP [16], SecretomeP [17], TargetP [18,19] and TMHMM [20,21]. The SignalP software package (version 4.1, http://www.cbs.dtu.dk/services/SignalP/) was used for identifying classical secretory proteins. All putative *D. gallinae* proteins which were not classified to contain signal peptide cleavage sites were further analyzed with SecretomeP (version 2.0, http://www.cbs.dtu.dk/services/SecretomeP/) for predicting non-classical secreted proteins. To limit false positive results the neural network (NN) score of ≥0.9 was set as described by Garg and Ranganathan [12]. To include only truly secreted *D. gallinae* proteins in subsequent analyses, proteins predicted to be secreted by either of the above mentioned software analyses were subsequently scanned for the presence of mitochondrial sequences by TargetP (version 1.1, http://www.cbs.dtu.dk/services/TargetP/) and transmembrane helices by TMHMM (version 2.0, http://www.cbs.dtu.dk/services/TMHMM/). Protein sequences identified to be of mitochondrial origin or exhibiting transmembrane helices were excluded from the “secreted” data set. Prediction of *D. gallinae* pTM proteins was carried out separately by scanning the putative *D. gallinae* protein sequences with TMHMM.

### Identification of protein homologs

For identifying homologous proteins, pES and pTM proteins were BLASTed (BLASTP) against the non-redundant (nr) database using the Blast2Go (b2g) software suite [22,23]. E-value cut-off was set at 1.0E-6.

### Functional annotation

Supported by b2g, *D. gallinae* pES and pTM proteins were functionally mapped to Gene Ontology terms [24,25] and annotated by setting default parameters (E-Value-Hit-Filter: 1.0E-6; Annotation cut-off: 55; GO weight: 5; Hsp-Hit Coverage cut-off: 0). Additionally, pES and pTM proteins were associated to protein families, domains and functional sites through InterProScan [26]. InterProScan integrated the following protein signature data bases: BlastProDom, FPrintScan, HMM-PIR, HMM-Pfam, HMM-Smart, HMM-Tigr, ProfileScan, Pattern Scan, Superfamily, Gene3D and HMM-Panther. pES and pTM proteins were subsequently passed to KOBAS2.0 [27] to identify statistically enriched related KO (KEGG Orthology) terms and KEGG pathways [28]. KOBAS was also used for KEGG gene mapping to identify pathways which are shared with the black-legged (deer) tick *Ixodes scapularis* as an example of a species belonging to the super order Parasitiformes. *I. scapularis* was chosen since its genome data are available [The *Ixodes scapularis* Genome project, http://extension.entm.purdue.edu/igp/; [29]].

## Results

### Predicted *D. gallinae* secretome and transmembranome size

Of the 55,129 putative *D. gallinae* protein sequences [15], a number of 2,935 sequences (5.3%) were predicted to contain a signal peptide cleavage site by SignalP. Of the remaining sequences, SecretomeP classified 1,450 protein sequences (2.6%) as non-classical secreted proteins. The putative 4,385 secreted proteins were parsed to TargetP and TMHMM resulting in 341 protein sequences (7.8%) predicted to be of mitochondrial origin and 953 protein sequences (21.7%) predicted to contain transmembrane helices. Potential mitochondrial and transmembrane proteins were excluded from the data set resulting in 3,091 *D. gallinae* pES protein sequences, representing 5.6% of the putative protein dataset. pTM protein prediction by TMHMM resulted in 7,361 (13.4%) sequences out of the 55,129 *D. gallinae* putative protein sequences.

### pES and pTM protein identification

**Figure 1:**
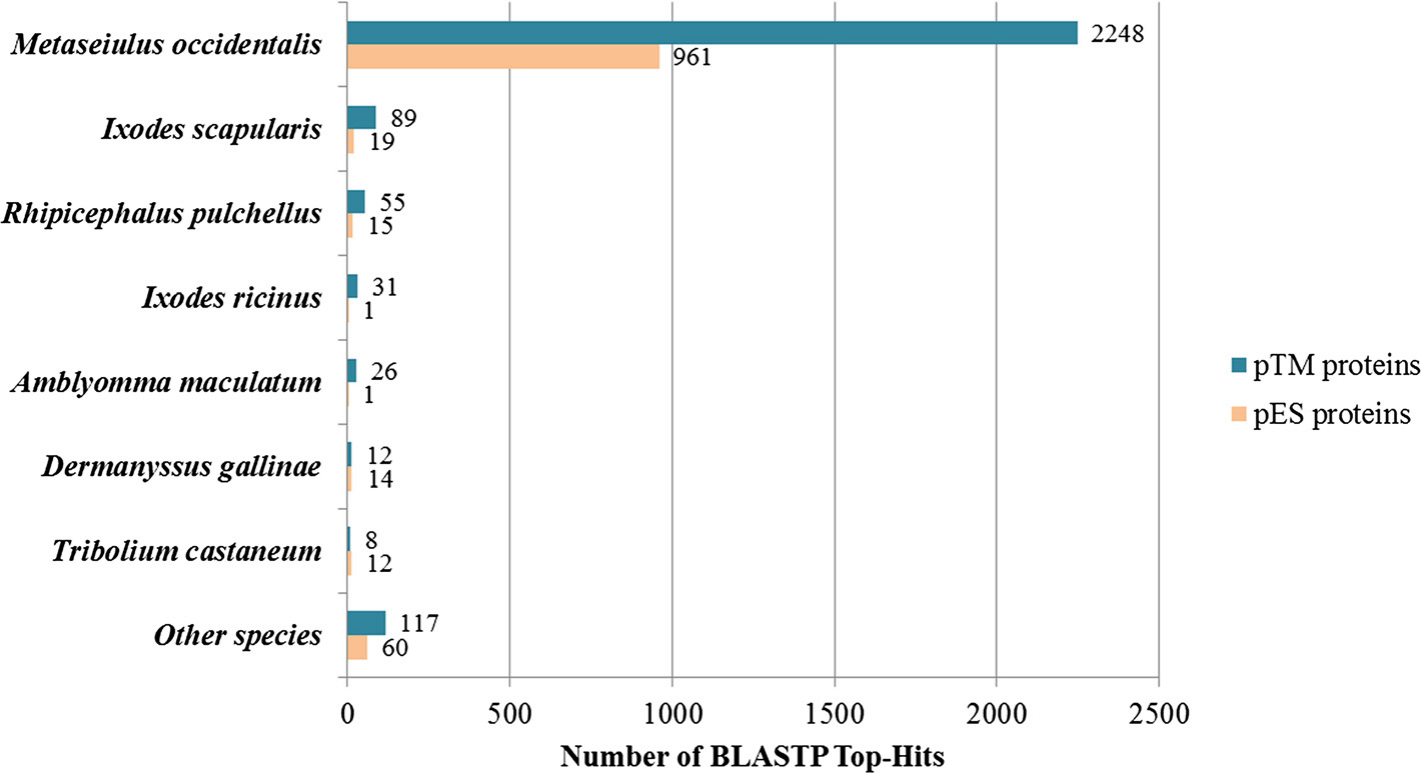
**BLASTP top-hit species distribution of *D. gallinae *pES and pTM protein sequences.**

Of the 3,091 *D. gallinae* pES protein sequences, a total of 1,083 (35.0%) showed significant BLASTP matches with proteins deposited in the GenBank database. A percentage of 88.7% (961 sequences) of *D. gallinae* pES proteins matched with proteins of the phytoseiid predatory mite *Metaseiulus occidentalis* followed with a considerably lower number of sequences by the tick species *I. scapularis* and *Rh. pulchellus* (Figure 1). *D. gallinae* pES proteins were identified as proteases [e.g. cysteine proteases such as cathepsins (45 sequences) or legumains (11 sequences)], cuticle proteins (27 sequences), salivary proteins (24 sequences), mucins (5 sequences), vitellogenins (4 sequences) and many more. About 20% of pES proteins represented uncharacterized or hypothetical/predicted protein homologs. An overview of BLASTP top-hits is given in Table 1, whereas detailed BLASTP results are listed in Additional file 1.

**Table 1:** **Top 30 BLASTP matches of *D. gallinae *pES proteins**

**Hit description**	**Species**	**Hit accession**	**No. of sequences**
Predicted: uncharacterized protein LOC100904214	*M. occidentalis*	XP_003739349	15
Predicted: uncharacterized protein LOC100908413	*M. occidentalis*	XP_003743523	11
Legumain-like protease precursor	*M. occidentalis*	XP_003746513	10
Cathepsin l-like	*M. occidentalis*	XP_003747496	10
Cathepsin l-1	*D. gallinae*	CCC33064	9
TBC1 domain family member 10A-like	*M. occidentalis*	XP_003737545	8
Predicted: uncharacterized protein LOC100898836	*M. occidentalis*	XP_003742669	8
Predicted: uncharacterized protein LOC100906884	*M. occidentalis*	XP_003743278	8
Predicted: cathepsin L-like	*M. occidentalis*	XP_003743816	8
Predicted: cuticle protein 10.9-like	*M. occidentalis*	XP_003744493	8
Predicted: uncharacterized protein LOC100900702	*M. occidentalis*	XP_003746592	8
Predicted: uncharacterized protein LOC100900705	*M. occidentalis*	XP_003747300	8
Predicted: uncharacterized protein LOC100905853	*M. occidentalis*	XP_003739512	7
Predicted: phospholipase A2-like	*M. occidentalis*	XP_003747592	7
Predicted: uncharacterized protein LOC100900008	*M. occidentalis*	XP_003737797	6
Chymotrypsin-like elastase family member 2A-like	*M. occidentalis*	XP_003740761	6
Predicted: soluble calcium-activated nucleotidase 1-like	*M. occidentalis*	XP_003741943	6
Predicted: cathepsin L-like	*M. occidentalis*	XP_003738729	5
Predicted: peroxidasin-like	*M. occidentalis*	XP_003743011	5
Predicted: peroxiredoxin-4-like	*M. occidentalis*	XP_003743456	5
Predicted: probable serine carboxypeptidase CPVL-like isoform 1	*M. occidentalis*	XP_003748333	5
Predicted: probable serine carboxypeptidase CPVL-like isoform 2	*M. occidentalis*	XP_003748334	5
Secreted salivary gland	*I. persulcatus*	BAH09304	4
Cathepsin D-1	*D. gallinae*	CCC33063	4
Predicted: uncharacterized protein LOC100903770	*M. occidentalis*	XP_003737317	4
Predicted: ferritin, liver middle subunit-like isoform 1	*M. occidentalis*	XP_003737398	4
Predicted: cuticle protein 10.9-like	*M. occidentalis*	XP_003739044	4
Predicted: frizzled-4-like	*M. occidentalis*	XP_003740054	4
Predicted: epidermal growth factor receptor-like	*M. occidentalis*	XP_003740683	4
Predicted: uncharacterized protein LOC100905037	*M. occidentalis*	XP_003740704	4

BLASTP homology search of the 7,361 pTM protein sequences revealed 2,586 (35.1%) matches with published protein sequences. Of these, 2,248 (86.9%) were homologous to *M. occidentalis* (cf. Figure 1). Besides hypothetical and uncharacterized proteins, representing about 15% of sequence homologs, ion channels, receptors and several transporters [e.g. ABC transporter (12 sequences), glucose (12 sequences), amino acid (44 sequences), protein (12 sequences), zinc (24 sequences) or ion transporter (39 sequences)] could be identified. A large part of *D. gallinae* pTM protein sequences were assigned to ninjurin-like proteins (96 sequences). Furthermore, proteins of the mite’s neuronal network such as GABA (2 sequences), glutamate (31 sequences) or acetylcholine (13 sequences) receptors and transporter (GABA: 11 sequences, glutamate: 4 sequences, acetylcholine: 2 sequences) were identified (Table 2 and Additional file 2).

**Table 2:** **Top 30 BLASTP matches of *D. gallinae *pTM proteins**

**Hit description**	**Species**	**Hit accession**	**No. of sequences**
Predicted: ninjurin-2-like	*M. occidentalis*	XP_003737707	96
Predicted: elongation of very long chain fatty acids protein 7-like	*M. occidentalis*	XP_003739733	50
Predicted: nose resistant to fluoxetine protein 6-like	*M. occidentalis*	XP_003740562	38
Hypothetical protein	*Rh. pulchellus*	JAA57419	28
Hypothetical protein	*Rh. pulchellus*	JAA54392	21
Predicted: steroid 17-alpha-hydroxylase/17,20 lyase-like	*M. occidentalis*	XP_003742972	18
Predicted: adipocyte plasma membrane-associated protein-like	*M. occidentalis*	XP_003748317	16
Predicted: cytochrome b561-like	*M. occidentalis*	XP_003737194	16
Predicted: voltage-dependent calcium channel type A subunit alpha-1-like	*M. occidentalis*	XP_003743405	14
Predicted: uncharacterized protein LOC100906895	*M. occidentalis*	XP_003747421	13
Predicted: solute carrier family 22 member 6-like	*M. occidentalis*	XP_003744619	13
hypothetical protein	*A. maculatum*	AEO35694	12
Predicted: canalicular multispecific organic anion transporter 1, partial	*M. occidentalis*	XP_003738712	12
Predicted: uncharacterized protein LOC100907708	*M. occidentalis*	XP_003742569	12
integral membrane protein, putative	*I. scapularis*	XP_002412964	12
Predicted: excitatory amino acid transporter 4-like	*M. occidentalis*	XP_003737125	11
Predicted: uncharacterized protein LOC100904062	*M. occidentalis*	XP_003737318	11
Predicted: lysophospholipid acyltransferase 7-like	*M. occidentalis*	XP_003746004	10
Predicted: PRA1 family protein 3-like	*M. occidentalis*	XP_003739732	10
Predicted: uncharacterized protein LOC100909236	*M. occidentalis*	XP_003746970	10
Predicted: protein transport protein Sec61 subunit gamma-like	*M. occidentalis*	XP_003742336	10
Putative lipid exporter abca1, partial	*Rh. pulchellus*	JAA63751	9
Predicted: putative sodium-coupled neutral amino acid transporter 7-like	*M. occidentalis*	XP_003744784	9
Predicted: vacuolar ATPase assembly integral membrane protein VMA21-like	*M. occidentalis*	XP_003744117	9
Predicted: aquaporin-10-like	*M. occidentalis*	XP_003745025	8
Predicted: aldehyde dehydrogenase, dimeric NADP-preferring-like	*M. occidentalis*	XP_003746957	8
Putative caax prenyl protease 1 log danio rerio zinc metallopeptidase ste24	*I. ricinus*	JAA68353	8
Predicted: membrane-bound O-acyltransferase domain-containing protein 2-like	*M. occidentalis*	XP_003747942	8
Predicted: NADH dehydrogenase [ubiquinone] 1 beta subcomplex subunit 5, mitochondrial-like	*M. occidentalis*	XP_003738022	8
Predicted: patched domain-containing protein 3-like	*M. occidentalis*	XP_003747353	8

### Functional annotation of *D. gallinae* pES and pTM proteins

**Figure 2:**
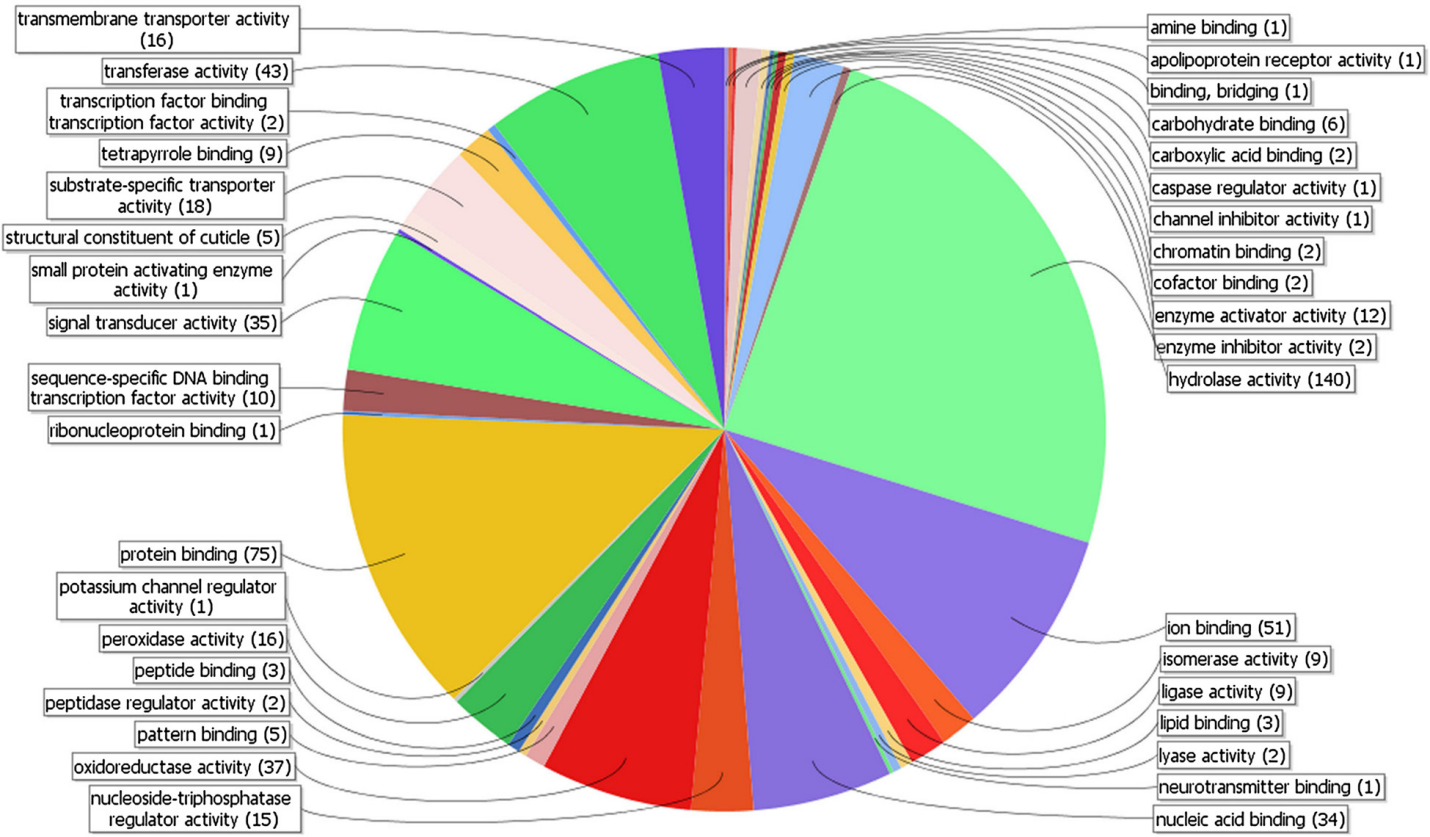
**Molecular function ontology distribution of *D. gallinae *pES proteins on third level subcategory.**

Functional annotation of *D. gallinae* pES and pTM proteins was based on Gene Ontology terms and assignment of protein families, protein domains and functional sites. Of the 3,091 *D. gallinae* pES proteins, 448 were assigned to 1,946 GO terms divided into 870 GO terms originating from the GO domain Biological Process, 383 GO terms from the Cellular Component domain and 693 GO terms from the Molecular Function domain (Additional file 3). Of the latter, 574 GO terms could be assigned to a third level subcategory (Figure 2), whereby the term hydrolase activity represented with 140 annotations - e.g. cathepsin L, cathepsin K, legumain or secreted mucin - nearly one fourth of assigned GO terms. This term originates together with the terms isomerase activity (9 annotations), ligase activity (9), lyase activity (2), oxidoreductase activity (37), small proteins activating enzyme activity (1) and transferase activity (43) from the parental GO term catalytic activity, which included 42.0% of all Molecular Function Ontology terms assigned to a third level subcategory. The second largest share was contributed to by the parental term binding (34.2%), represented amongst others by the third level subcategory terms protein binding (75 annotations), peptide binding (3), nucleic acid binding (34), carbohydrate binding (6) and lipid binding (3).

**Figure 3:**
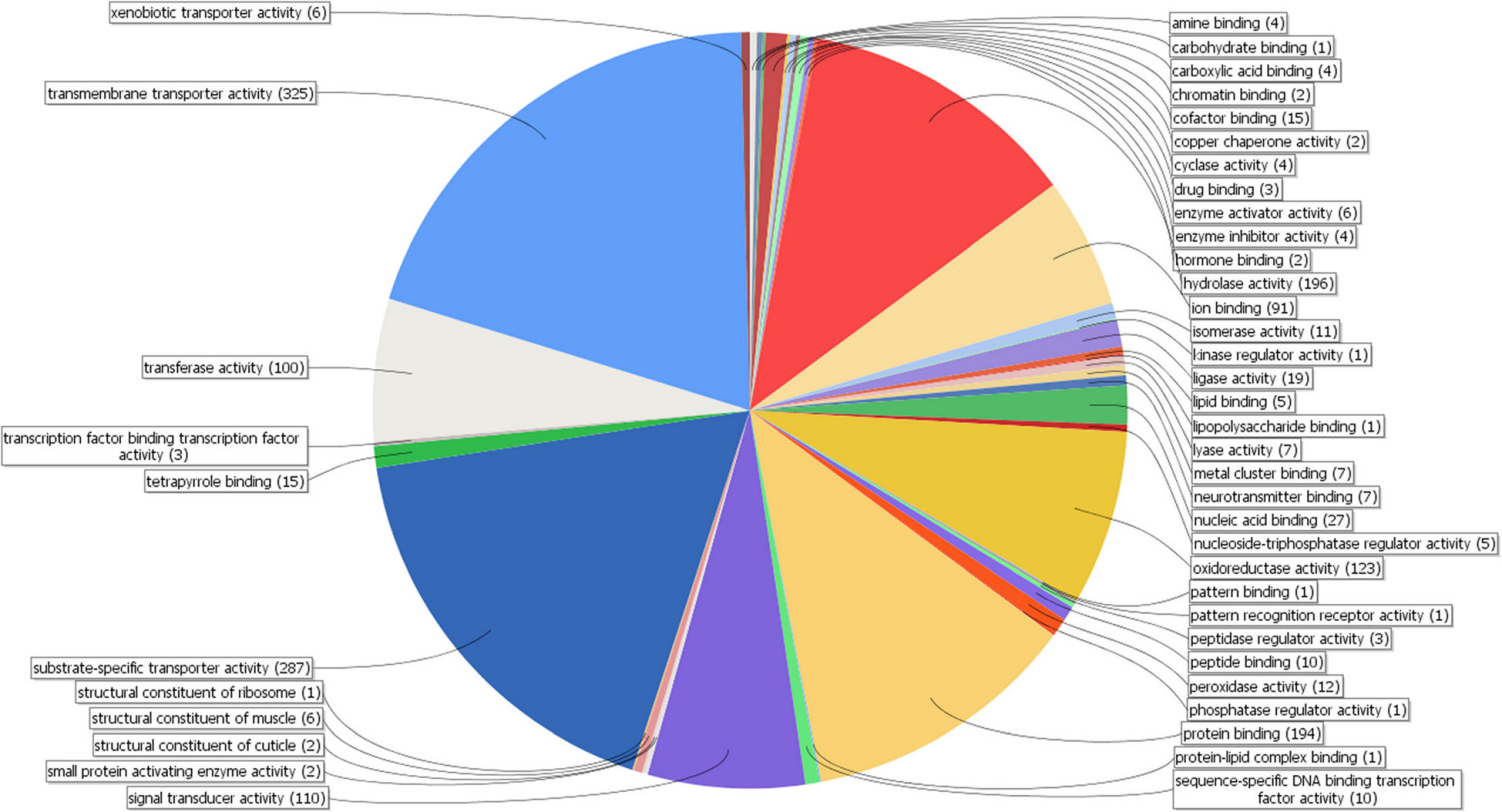
**Molecular function ontology distribution of *D. gallinae *pTM proteins on third level subcategory.**

Of the total 7,361 *D. gallinae* pTM proteins, 1,253 were mapped to 6,610 GO terms originating from 3,097 Biological Processes terms, 1,720 Molecular Function terms and 1,793 Cellular Component terms (Additional file 4). On a third level subcategory, 1,637 GO terms were assigned to the Molecular Function domain (Figure 3), the largest part of which (325 annotations) being mapped to transmembrane transporter activity, followed by substrate-specific transporter activity (287) and hydrolase activity (196).

InterPro annotation of pES protein sequences resulted in 531 different assigned protein domains and families. A major part of *D. gallinae* pES proteins represented proteases assumed to be involved in proteolytic digestion. Another significant share was Immunoglobin-like proteins or subtypes involved in many functions such as cell-cell recognition, cell-surface receptors, muscle structure and immune functions. InterProScan of pTM protein sequences revealed 859 protein domains and families of which ninjurin was the most frequently occurring domain. A further large share of pTM protein sequences were assigned to different transporter systems such as ion channels, ABC transporter or symporter, and cytochrome P450 domains. The most frequently occurring protein domains and families of pES and pTM proteins of *D. gallinae* are shown in Table 3.

**Table 3:** **Top 25 protein domains and families of *D. gallinae *pES and pTM proteins**

**pES proteins**		**pTM proteins**	
**InterPro code and description**	**No. of pES proteins (%)**	**InterPro code and description**	**No. of pTM proteins (%)**
IPR013128 Peptidase C1A, papain	42 (1.36)	IPR007007 Ninjurin	97 (1.32)
IPR013201 Proteinase inhibitor I29, cathepsin propeptide	41 (1.33)	IPR016196 Major facilitator superfamily domain, general substrate transporter	56 (0.76)
IPR013783 Immunoglobulin-like fold	34 (1.10)	IPR002076 GNS1/SUR4 membrane protein	53 (0.72)
IPR009003 Peptidase cysteine/serine, trypsin-like	32 (1.04)	IPR016040 NAD(P)-binding domain	49 (0.67)
IPR001254 Peptidase S1/S6, chymotrypsin/Hap	31 (1.00)	IPR001128 Cytochrome P450	46 (0.62)
IPR000618 Insect cuticle protein	27 (0.87)	IPR005821 Ion transport domain	44 (0.60)
IPR007110 Immunoglobulin-like	25 (0.81)	IPR002401 Cytochrome P450, E-class, group I	34 (0.46)
IPR000668 Peptidase C1A, papain C-terminal	24 (0.78)	IPR017452 GPCR, rhodopsin-like, 7TM	30 (0.41)
IPR001314 Peptidase S1A, chymotrypsin-type	24 (0.78)	IPR000175 Sodium:neurotransmitter symporter	29 (0.39)
IPR018114 Peptidase S1/S6, chymotrypsin/Hap, active site	23 (0.74)	IPR002198 Short-chain dehydrogenase/reductase SDR	29 (0.39)
IPR000169 Cysteine peptidase, cysteine active site	22 (0.71)	IPR013783 Immunoglobulin-like fold	29 (0.39)
IPR012336 Thioredoxin-like fold	18 (0.58)	IPR000276 GPCR, rhodopsin-like	27 (0.37)
IPR025661 Cysteine peptidase, asparagine active site	18 (0.58)	IPR002347 Glucose/ribitol dehydrogenase	27 (0.37)
IPR013098 Immunoglobulin I-set	14 (0.45)	IPR001140 ABC transporter, transmembrane domain	24 (0.33)
IPR013781 Glycoside hydrolase, catalytic domain	14 (0.45)	IPR017940 ABC transporter, integral membrane type 1	24 (0.33)
IPR017853 Glycoside hydrolase, superfamily	14 (0.45)	IPR020846 Major facilitator superfamily domain	24 (0.33)
IPR002007 Heme peroxidase, animal	13 (0.42)	IPR017972 Cytochrome P450, conserved site	23 (0.31)
IPR010255 Heme peroxidase	13 (0.42)	IPR011527 ABC transporter, transmembrane domain, type 1	22 (0.30)
IPR003598 Immunoglobulin subtype 2	12 (0.39)	IPR011042 Six-bladed beta-propeller, TolB-like	21 (0.29)
IPR003599 Immunoglobulin subtype	12 (0.39)	IPR013099 Ion transport 2	21 (0.29)
IPR001096 Peptidase C13, legumain	11 (0.36)	IPR006201 Neurotransmitter-gated ion-channel	20 (0.27)
IPR001563 Peptidase S10, serine carboxypeptidase	11 (0.36)	IPR004299 Membrane bound O-acyl transferase, MBOAT	19 (0.26)
IPR013083 Zinc finger, RING/FYVE/PHD-type	11 (0.36)	IPR011701 Major facilitator superfamily	18 (0.24)
IPR002018 Carboxylesterase, type B	10 (0.32)	IPR005828 General substrate transporter	17 (0.23)
IPR011009 Protein kinase-like domain	10 (0.32)	IPR018108 Mitochondrial substrate/solute carrier	17 (0.23)

KEGG pathway mapping revealed 252 pES proteins to be involved in 180 pathways. The most frequently assigned pathway was lysosome, followed by antigen processing and presentation. Phagosome was the third most frequent pathway. Mapping of *D. gallinae* pES protein sequences against *I. scapularis* KEGG GENES resulted in assigning 133 pES proteins to 60 pathways. The analysis revealed lysosome as the most frequent and phagosome as the third most frequent pathway as well, whereas the second most frequent pathway was represented by metabolic pathways. The top 15 KEGG pathways as well as *I. scapularis* pathways of pES protein sequences are listed in Table 4. A total of 611 pTM protein sequences were found to be involved in 210 KEGG pathways with protein processing in endoplasmic reticulum being the most frequently occurring pathway, followed by lysosome and Huntington’s disease (cf. Table 5). Furthermore, pTM protein sequences were mapped to neuronal processes such as neuroactive ligand-receptor interaction or glutamatergic synapse. KEGG Gene mapping of *D. gallinae* pTM protein sequences to *I. scapularis* revealed 331 pTM protein sequences to be involved in 60 pathways. The most frequently shared assigned pathway were metabolic pathways followed by protein processing in endoplasmic reticulum and lysosome.

**Table 4:** **Top 15 KEGG pathways of *D. gallinae *pES proteins**

**KEGG pathway**	**No. of sequences (%)**	**No. of enzymes**	***I. sapularis *KEGG pathway**	**No. of sequences (%)**	**No. of NCBI Gene ID’s**
ko04142 Lysosome	58 (1.88)	17	isc04142 Lysosome	41 (1.33)	12
ko04612 Antigen processing and presentation	37 (1.20)	5	isc01100 Metabolic pathways	26 (0.84)	21
ko04145 Phagosome	29 (0.94)	7	isc04145 Phagosome	18 (0.58)	6
ko05323 Rheumatoid arthritis	22 (0.71)	4	isc04141 Protein processing in endoplasmic reticulum	17 (0.55)	12
ko04141 Protein processing in endoplasmic reticulum	19 (0.61)	14	isc04310 Wnt signaling pathway	9 (0.2)	6
ko00230 Purine metabolism	16 (0.52)	7	isc00230 Purine metabolism	8 (0.26)	5
ko00240 Pyrimidine metabolism	16 (0.52)	7	isc00240 Pyrimidine metabolism	8 (0.26)	5
ko05166 HTLV-I infection	14 (0.45)	8	isc00511 Other glycan degradation	6 (0.19)	3
ko04310 Wnt signaling pathway	12 (0.39)	7	isc03008 Ribosome biogenesis in eukaryotes	6 (0.19)	3
ko05200 Pathways in cancer	12 (0.39)	6	isc03018 RNA degradation	5 (0.16)	2
ko04510 Focal adhesion	11 (0.36)	7	isc04120 Ubiquitin mediated proteolysis	4 (0.13)	4
ko00860 Porphyrin and chlorophyll metabolism	9 (0.29)	3	isc00600 Sphingolipid metabolism	4 (0.13)	3
ko04010 MAPK signaling pathway	9 (0.29)	6	isc03013 RNA transport	4 (0.13)	3
ko04972 Pancreatic secretion	9 (0.29)	7	isc04070 Phosphatidylinositol signaling system	4 (0.13)	3
ko04020 Calcium signaling pathway	8 (0.26)	5	isc03015 mRNA surveillance pathway	4 (0.13)	2

**Table 5:** **Top 15 KEGG pathways of *D. gallinae *pTM proteins**

**KEGG pathway**	**No. of sequences (%)**	**No. of enzymes**	***I. sapularis *KEGG pathway**	**No. of sequences (%)**	**No of NCBI Gene ID’s**
ko04141 Protein processing in endoplasmic reticulum	60 (0.81)	25	isc01100 Metabolic pathways	104 (1.43)	52
ko04142 Lysosome	52 (0.71)	15	isc04141 Protein processing in endoplasmic reticulum	54 (0.73)	21
ko05016 Huntington’s disease	52 (0.71)	21	isc04142 Lysosome	50 (0.68)	13
ko00190 Oxidative phosphorylation	48 (0.65)	24	isc00190 Oxidative phosphorylation	39 (0.53)	16
ko04020 Calcium signaling pathway	44 (0.6)	18	isc04145 Phagosome	23 (0.31)	8
ko05010 Alzheimer’s disease	43 (0.58)	21	isc04080 Neuroactive ligand-receptor interaction	22 (0.30)	11
ko04080 Neuroactive ligand-receptor interaction	42 (0.57)	24	isc03060 Protein export	20 (0.27)	7
ko04724 Glutamatergic synapse	42 (0.57)	16	isc00564 Glycerophospholipid metabolism	19 (0.26)	7
ko05012 Parkinson’s disease	41 (0.56)	16	isc00510 N-Glycan biosynthesis	18 (0.24)	10
ko02010 ABC transporters	34 (0.46)	15	isc04146 Peroxisome	16 (0.22)	6
ko04972 Pancreatic secretion	34 (0.46)	14	isc04310 Wnt signaling pathway	13 (0.18)	6
ko00564 Glycerophospholipid metabolism	32 (0.43)	13	isc00600 Sphingolipid metabolism	11 (0.15)	6
ko04970 Salivary secretion	30 (0.41)	13	isc04120 Ubiquitin mediated proteolysis	11 (0.15)	6
ko04145 Phagosome	27 (0.37)	12	isc01040 Biosynthesis of unsaturated fatty acids	10 (0.14)	4
ko04723 Retrograde endocannabinoid	28 (0.38)	14	isc00020 Citrate cycle (TCA cycle)	9 (0.12)	4

## Discussion

The secretome is a part of the proteome of an organism and includes all proteins secreted by the cell including those of the extracellular matrix, proteins shed from the cell membrane and vesicle proteins like microsomal vesicles [12,30-32]. Transmembrane proteins are a group of membrane proteins containing subunits exposed on both sides of a cell membrane. They compose approximately 30% of a typical genome and are involved in many important biological processes including cell signaling, transport of membrane-impermeable molecules and cell recognition [33]. As the present *D. gallinae* secretome as well as transmembranome predictions and analyses were based on putative proteins of transcriptome data including all developmental stages of starved as well as fed whole body poultry red mites [15], a broad spectrum of pES and pTM proteins originating from different metabolic pathways was expected. About one fifth (19%) of *D. gallinae* putative proteins was assigned to the mite’s secretome or transmembranome, divided into 5.6% (3,091) secreted and 13.4% (7,361) transmembrane proteins, respectively. Of these predicted proteins, ~35.0% showed significant matches with known protein sequences, with the predatory phytoseiid mite *M. occidentalis* being the top-1 hit of 89% *D. gallinae* pES proteins and 87% pTM proteins. This was not unexpected since both mite species share a close relationship and furthermore, the genome as well as the transcriptome of *M. occidentalis* is sequenced and available in the common data bases [34,35]. The share of 65% pES and pTM proteins which could not be identified via BLAST and thus were categorized as “novel” are either parasite specific proteins or even unique for the poultry red mite, underlining the importance of further research on protein characterization.

The major part of pES proteins were identified as hydrolases and of these, a large share was cysteine proteases. Cysteine proteases are important in different biochemical and physiological processes of arthropods like embryogenesis [36-42]. The arthropod embryo needs a lot of nutrients during its development. It obtains its nutrition from egg reserve material consisting of amino acids, carbohydrates and lipids stored in yolk granules. To make these nutrients available, enzymatic machinery is needed [39]. Degradation of the yolk protein vitellin is triggered by acidification of the yolk granules, activating as a consequence cysteine proteinases like cathepsin L and B [37-39,43] and aspartic proteinases like cathepsin D [44]. Besides embryogenesis, these cathepsins may be essential in the proteolytic digestion of the mite’s blood meal. This might be extrapolated from the well-studied blood digestion in the closely related ticks [45-49] since both, ticks and *D. gallinae* share anatomic similarity of the intestinal tract belonging to the Parasitiformes type. As summarized by Horn *et al.*[45], the primary cleavage of hemoglobin in the hard tick *I. ricinus* is accomplished by the endopeptidase cathepsin D, which is supported by the catalytic activity of cathepsin L and legumain. These three proteases represent 6 of the top-30 BLASTP matches of the poultry red mite’s secretome (cf. Table 1). The protein family and domain analysis of *D. gallinae* pES proteins revealed a high proportion of cysteine peptidases C1A, papain (IPR013128: 42 pES sequences; IPR000668: 22 pES sequences) and C13, legumain (IPR001096: 11 pES sequences). Santamaria *et al.*[50] compared the number of different protein families of the phytophageous mite *Tetranychus urticae* to different arthropod species (genome data of 10 insects, 1 crustacean and 1 tick) and found that C1A papain-like peptidases are common in all species, whereas a high number of C13 legumain-like peptidases (19 genes) was found in *T. urticae* mites compared to the other arthropods. The eleven predicted legumain-like peptidases in *D. gallinae* suggest that extensive expansion of this protein family amongst mites might not be unusual. KEGG pathway mapping predicted pES proteins to be most frequently located in the lysosome in both pathways of all organisms and the tick *I. scapularis* alone. This might result from the large number of *D. gallinae* pES proteins identified as proteases like cathepsins, which may act as endopeptidases in the lysosome [51]. As in mites, digestive processes of ticks take place in the acid endosomal/ lysosomal vesicles of gut epithelia cells, contrary to other blood feeding arthropods [52,53].

With the identification and analyses of different blood feeding-induced molecules of ticks, an antigen against the cattle tick *Rhipicephalus microplus* (formerly *Boophilus microplus*) was found [54] leading to successful vaccine development against this tick (TickGARD plus™/Gavac™). The antigen Bm86 is a membrane-bound glycoprotein of the tick’s intestinal tract [54,55]. As a gut protein, Bm86 is not part of the normal host-tick interaction and therefore does not stimulate an immunological response under normal circumstances. However, vaccination with this “concealed” or “hidden” antigen induced an immunological response of the cattle host consequently damaging the blood feeding tick [56,57]. When considering potential feeding-induced predicted *D. gallinae* pTM and pES molecules as concealed antigens, a broad range of new vaccine candidates against the poultry red mite is provided by the present study, e.g. legumains, chymotrypsins or cathepsins. Of these, cathepsin D and L were suggested by Bartley *et al.*[58] as part of a multi-component vaccine against the poultry red mite due to their efficiency in an *in vitro* feeding assay. Notably, BLASTP search of predicted *D. gallinae* pTM proteins did not result in a *Rh. microplus* Bm86 hit amongst the top-20 BLAST Hits of each *D. gallinae* pTM protein sequence. This may indicate that no Bm86 homologue is present in the poultry red mite, although as this search was based on transcriptomic data it could be present albeit at low abundance or alternatively it may not be expressed. Specific analysis of the mite’s gut transcripts could test this hypothesis. However, in contrast to ticks, dissection of *D. gallinae*’s gut is not possible [59] due to its small size. This is, depending on the developmental stage, about 0.39-1 mm in length and 0.26-0.64 mm in width [60]. Thus, estimating gut-derived pTM proteins remains complicated as proteases and other molecules suggested to be involved in blood digestion may also play a role in other processes. Besides “hidden” antigens “exposed” antigens like salivary proteins are also discussed as vaccine candidates against ticks [57,61] as they may act as immunomodulators [62,63]. Therefore, further research on the 24 predicted salivary proteins of *D. gallinae* could also promote vaccine development.

The BLASTP top-hit of the *D. gallinae* transmembranome is represented by a ninjurin-2-like protein. In mammals, ninjurin acts as an adhesion molecule which is induced by nerve injury and promotes axonal growth but is also expressed in a number of other tissues, predominantly in epithelial cells [64]. Correlation of ninjurin upregulation with wounding incidents was also shown in adult *Drosophila melanogaster* flies [65] and in the mosquito *Anopheles gambiae* ninjurin is suggested to play an important role in innate immune response, probably in signaling and cell communication [66]. Whether the abundance of ninjurin-like proteins amongst the *D. gallinae* pTM proteins results from the preparation steps (immobilized living mites were collected via forceps which might have injured the mites), from immune functions or from completely different, hitherto unknown functions in mites remains unclear. Another interesting and unexpected pTM protein homolog was the third most common BLASTP top-hit, which is predicted as being nose resistant to fluoxetine protein 6-like (*nrf-6*). This transmembrane protein, which acts in the intestine of *Caenorhabditis elegans* and confers sensitivity to the antidepressant fluoxetine (Prozak), is suggested to play a role in drug or yolk transport across membranes [67,68]. The *D. melanogaster beltless* (*blt*) gene shares homologies with the *C. elegans nrf-6* and is crucial during oogenesis and embryogenesis, but also expressed in the adult nervous system and thus suggested to be important for neuronal functioning [69]. In mites, function and localization of genes involved in neurological processes is largely unknown. Research work is needed to characterize *D. gallinae* pTM proteins involved in neurological processes, since - if one extrapolates from the mode of action of most acaricides - they could represent promising candidates for identifying new drug targets against poultry red mites.

## Conclusion

The present study is the first of its kind analyzing an acarid secretome as well as transmembranome *in silico*. It provides valuable insights into a pool of *D. gallinae* proteins, which might be relatively easily accessible candidates for drugs or immunological components like antibodies and thus represent potential drug targets or vaccine candidates. In particular, pES or pTM proteins suggested to be involved in blood feeding and digestion or neurological processes provide a promising basis for further research on new intervention strategies against *D. gallinae*, which is considered one of the most serious pests of poultry. Nevertheless, future studies on the proteomic level are highly desirable to confirm the predicted secretome and transmembranome of the red poultry mite as *in silico* analyses rely on the use of algorithms to identify sequence features (i.e. signal peptide motifs) and thus do not necessarily accurately reflect the entire biological truth.

## Competing interests

The authors declare that they have no competing interests.

## Authors’ contributions

CS conceived and designed the research plan, coordinated the study and participated in writing of the manuscript. SS and WQ performed bioinformatic analyses. SS, WQ, LP and CS analyzed and interpreted the data. SS drafted the manuscript. All authors read and approved the final manuscript.

## Supplementary Material

Additional file 1 **BLASTP top-hits of *D. gallinae *pES proteins.** Click here for file

Additional file 2 **BLASTP top-hits of *D. gallinae *pTM proteins.** Click here for file

Additional file 3 **Gene Ontology term distribution of *D. gallinae *pES proteins.** Click here for file

Additional file 4 **Gene Ontology term distribution of *D. gallinae *pTM proteins.** Click here for file
